# Visual expertise modulates baseline brain activity: a preliminary resting-state fMRI study using expertise model of radiologists

**DOI:** 10.1186/s12868-022-00707-x

**Published:** 2022-04-12

**Authors:** Ting Zhang, Minghao Dong, Hongmei Wang, Rui Jia, Fu Li, Xiaoli Ni, Chenwang Jin

**Affiliations:** 1 School of Humanities and Social Science, Xi’an Jiaotong University, Xi’an, China; 2 College of Tourism & Landscape Architecture, Guilin University of Technology, Guilin, China; 3 Engineering Research Center of Molecular and Neuro Imaging of Ministry of Education, School of Life Science and Technology, Xidian University, Xi’an, 710071 Shaanxi China; 4 Xi’an Key Laboratory of Intelligent Sensing and Regulation of Trans-Scale Life Information, School of Life Science and Technology, Xidian University, Xi’an, 710126 Shaanxi China; 5 Department of Medical Imaging, First Affiliated Hospital of Medical College, Xi’an Jiaotong University, Xi’an, China; 6 College Students Mental Health Education Center, Xi’an University of Technology, Xi’an, China; 7 Key Laboratory of Intelligent Perception and Image Understanding of Ministry of Education, School of Artificial Intelligence, Xidian University, Xi’an, China

**Keywords:** Visual expertise, Plasticity, Resting state fMRI, Radiologists

## Abstract

**Background:**

visual expertise and experience modulate evoked brain activity in response to training-related stimuli. However, few studies have considered how the visual experience is represented in the resting state brain activity. This study tried to investigate the way visual experience, i.e., visual recognition expertise, modulates baseline brain neuronal activity in the resting state using the model of radiologists.

**Methods:**

The amplitude of low-frequency (< 0.08 Hz) fluctuation (ALFF) was used as the metric of baseline brain activity and a visual expertise model of radiologists to investigated this question. The visual recognition skill enables them to accurately identify pathological information in medical images. After the behavior measurement, a cohort group of radiology interns (n = 22) and a group of matched layperson (n = 22) were selected for inclusion in the study. The resting state functional magnetic resonance imaging (fMRI) scans were performed for all of the subjects.

**Results:**

Higher ALFF in the right fusiform gyrus and the left orbitofrontal cortex were observed, and the ALFF in the fusiform gyrus was correlated with the intern radiologists’ behavioral expertise(all results corrected for multiple comparisons).

**Conclusions:**

Visual experience modulates the baseline brain activity in both high-level visual cortex and high-order cognitive cortex, indicating the engagement of both top-down and bottom-up facilitation. We provide a novel perspective to how visual experience modulated cortical brain activity by introducing the resting state changes. Also, we propose that our current study may provide novel ideas for the development of new training protocols in medical school.

## Background

Visual expertise, i.e. expertise in visual object recognition, refers to fine level visual discrimination of homogeneous stimuli, which is acquired through extensive visual experience within a given object category [1]. [2–6] Continuing effort has been expended to better understand[7] the neural substrate underlying such proficiency. Previous studies reported evoked brain activity in both visual system and high-order cognitive regions across the brain [8, 9] [10–13]

In the adult human brain, visual information processing is highly malleable with neural processing adapting to incoming information [14]. These experiences continually shape the spatial and temporal organization of cortical representations of stimuli [15]. In medical practice, the ability to make fine distinctions among visually similar stimuli is the primary basis of detecting and diagnosing disease for radiologists [7, 16]. Given their exceptional radiological-specific visual recognition skill, radiologists serve as a rare but important model to study visual expertise, [10, 11, 16, 17]. This perceptual specialty is acquired through intensive training during which hundreds of cases are reviewed [3, 6]. Recently, a few studies investigated the functional anatomy of visual expertise under tasks using the expertise model of radiologists [10–12, 18]. Haller et al. [11] and Ouellette et al. [18] observed activation in the ventral visual pathway, including the right fusiform gyrus (FG), and higher-order brain regions, such as the left inferior frontal gyrus in radiologists in differentiating X-ray films than novices. Harley et al. specifically investigated the visual pathway and reported engagement of FG when radiologists detected abnormalities in chest radiographs [10]. Bilalić et al. explicated stronger FG activation in response to radiological images [12]. In sum, available evidence supports neuronal plasticity at wide-spread cortical sites involved in the task [19].

Nevertheless, we propose that the information implanted in the resting data, as revealed in the intrinsic brain activity, is important in that (1) neuronal synchronization is encoded in spontaneous low-frequency fluctuations in the blood oxygen level-dependent (BOLD) signal [20, 21]; (2) spontaneous cortical activity plays an important role in the internal representations and maintaining the ongoing [22, 23], which are involved in the coding of previous experience [24, 25]; (3) experience-dependent neuroplastic changes shape the pattern of spontaneous activity within the resting brain [26, 27] and such alterations bear behavior significance [28–30]. Therefore, resting state brain activity is a new window to understand the neural substrate of expertise in the context of neural plasticity [29].

Among all the issues related to resting state spontaneous neuronal activity, the baseline brain activity is of particular significance. The baseline spontaneous neuronal activity reflects cortical excitability [21, 31], the alteration of which influences the strength of connection and connectomes-based analysis in resting fMRI studies [32, 33], as well as pattern of the spatial activation under task [34, 35]. Previous studies used the amplitude of low-frequency (< 0.08 Hz) fluctuation (ALFF) as the metric of brain intrinsic activity [35, 36]. Moreover, established evidence demonstrated that ALFF serves as an indicator of cortical excitability [37] and the volume of regional cerebral blood flow was correlated with ALFF in the brain region from the resting state data [38], therefore, ALFF was used to assess the intrinsic brain activity in this study.

Accordingly, in the current study, we evaluated the ALFF and a group of radiology interns (N = 22) after short-term radiological training in local hospital and a group of matched healthy layer-person to assess how radiological visual experience alters interns’ baseline brain activity. First, the level of recognition expertise in radiology was evaluated using radiological recognition behavioral tasks. Second, given previous learning experience modulates resting state activity [27, 28], we expected to see changes in the higher visual cortices and higher-order brain regions, which is supportive of higher visual abilities, i.e. visual pattern recognition. Third, we examined how the level of visual recognition expertise in radiology were related to ALFF alterations in radiologists. Given the paucity of studies focusing on the neural substrate in radiologists, we proposed that our study offers the first evidence on how radiological experience changes the brain representation in the resting state.

## Methods

This study was approved by the Ethical Committee of First Affiliated Hospital of Medical College subcommittee on Human Studies and was conducted in accordance with the Declaration of Helsinki.

### Experimental procedure

Given the scarcity of radiology interns, the matched non-expert control group (NECG) were recruited after the radiology interns group (RIG) were recruited. The radiologist interns were supposed to undertake B-scan ultrasonography, X-ray departments, rotations in MRI, and positron emission tomography-computed tomography (PET-CT) within 4 months in a randomized fashion. We managed to align all the participants’ training arrangement to starting from the X-ray department, which lasted for one month, after coordination with the hospital. For the current study, we only managed to collect the MRI data of RIG after rotation in the X-ray Department.

Basically, the RIG underwent the prescreening, MRI scan and behavioral measurement after one-month training in the X-ray department. Days before MRI data acquisition, the prescreening was conducted to ensure they were righted-handed by a face-to-face interview using questionaries [39]. The effect of visual expertise from other known domains (e.g., cars, chess, birds and mushrooms) was also excluded. MRI scanning was taken without telling the purpose of this study (elaborated in "MRI data acquisition" section), immediately after which behavior measurement was conducted (*fully elaborated in 2.3*). This arrangement minimized the possibility of directing subjects’ attention to the same content.

The subjects of the NECG were selected from the Control Subjects Database for Visual Expertise (CSDVE), which was set up for visual expertise studies by our group. The subjects from CSDVE had no previous experience in medical field, including experience in radiography and visual expertise from other known domains, i.e., cars, chess, birds and mushrooms. Basic demographical and behavioral information, such scores of handedness [39], level of education and level of face expertise [40] were collected beforehand and stored in the CSDVE. After the matched subjects were selected based on factor such as handedness, level of education and level of face expertise, the MRI scanning was conducted without telling subjects the purpose of this study (elaborated in "MRI data acquisition" section), succeeded by behavior measurement including Radiological Expertise Task (RET) and Cambridge Face Memory Test (CFMT) (*fully elaborated in 2.3*). Results of these two tasks were used for further analysis. Please note that the scores for the CFMT in the CSDVE were only used for subject selection, and the results of the CFMT after MRI scanning were included for data analysis.

### Subjects

The subjects of the current study consist of a cohort group of radiology interns and a group of matched layerperson as the control group. The level of expertise in radiology interns and in controls was evaluated in prescreening interviews (as explicated in the section of *Behavior measurement*). Twenty-two healthy, right-handed [39], radiology interns (11 males, mean age 23 ± 0.7 years (mean ± standard deviation, SD)] and 22 healthy non-expert control subjects, matched for sex, level of education and age (11 males, mean age 23 ± 0.5 years (mean ± SD)) were recruited. The RIG consisted of medical students on the undergraduate program in national medical schools following the same training protocol; the program was required to follow the same syllabus for students to be included. The subjects in the RIG underwent rotation in the X-ray department in the past 4 weeks, during which they reviewed 25–35 cases each day, six days a week. The mean duration of rotation was 26 ± 2.5 (mean ± SD) days. Each of them had a tutor providing clinically based support at the end of their daily practice. Students build a recorded portfolio of experience with a minimum of 600 cases as recorded in the Picture Archiving and Communication System (PACS) over the rotation period. Each case report of the radiology interns is matched for ‘degree of agreement’ against the decision of the tutor radiologist. During the rotation, the radiology interns were required to identify the pathologies in the X-ray films displayed on the screen, and complete the report; therefore, their experience was centered on interpreting X-ray images.

On the other hand, NECG consisted of 22 students. We also ensured that the control subjects had no known category of visual expertise by questionnaire, such as chess, cars, birds and mushrooms. No past or current neurological disorders, neuropsychological disorders or psychiatric disorders were reported and drugs or illegal medication before or during the study was taken for all subjects. All participants gave written informed consent after the experimental procedures were fully explained and had normal and corrected-to-normal vision when participating in tests outside the scanner and localizer scans inside the scanner. The radiological images used for pre-screening procedure and localizer scans were different.

### Behavior measurement

Viewing conditions were controlled by the exclusion of natural light. The same test banks were used in all the experiments for both RIG and NECG. The test was not started until the experimenter has confident that the protocol was fully understood by the observers after they repeated the whole procedure to the experimenter.

**Fig. 1:**
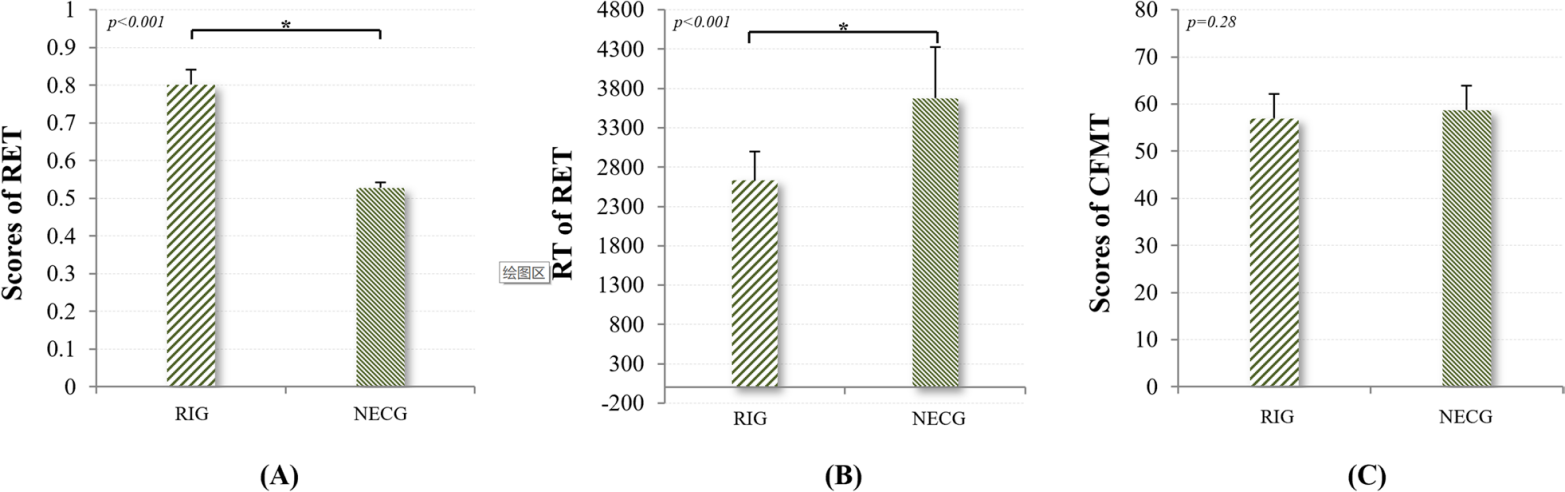
Results of behavior measurement for both groups. **A** The level of perceptual expertise in the domain of radiological images as assessed by the Radiological Expertise Task. The radiology interns group had a significantly larger AUC than the normal control group (*p*<0.001, Mann-Whitney test), indicating better visual recognition ability in radiological images; **B** Response time of both groups in the Radiological Expertise Task; **C** The level of perceptual expertise in the domain of faces as measured by the Cambridge Face Memory Test. *RET *Radiological Expertise Task, *RIG* radiology interns group, *NECG* normal control group, *CMFT* Cambridge Face Memory Test. *Indicates significant group differences (*p* < 0.05)

Given that perceptual expertise is highly domain specific [41], the behavior test for their visual expertise level should be specific to X-ray images. At the end of their rotation in the radiology department, participants were assessed by a practical examination of radiological anatomy and interpretation of X-ray images. Specifically, we developed a behavior task to measure subjects’ perceptual ability in medical imaging, namely the Radiological Expertise Task (RET) following guidance from the book “The Handbook of Medical Image Perception and Techniques” [42]. Participants’ training focused on radiological images in the first period of rotation; therefore, we selected 100 of standard chest images of adults (65 positive images and 35 negative images) from the X-ray image bank of the Department of Medical Imaging, First Affiliated Hospital of Medical College. The selected films were inspected for pathological appearance by 3 senior independent expert radiologists (with more than 10 years of radiological experience) and approved by confirmed radiological reports. The level of difficulty for judgement was assessed by the same 3 senior independent expert radiologists on the scale of 1 to 3. The positive images contained only one presence of disease. The portion for each level of difficulty is 55%, 30% and 15%, also with the factor of prevalence taken into consideration [4]. The observers were told that each X-ray images might contain zero or one single nodule and their task was to decide on a nodule’s presence and the confidence of their judgment. The observers were instructed to make decision for each image within 5 s using an in-house radiological behavior data collection system (Fig. 1A). All their input, i.e., their judgement of the presence or absence or the disease, the confidence in the judgement, and their response time, were recorded by in-house software (Chinese Software Patent NO. 2018SR036699, http://rsvp.dingdongyun.com/). Standard receiver operating characteristic (ROC) curve analysis was carried out to evaluate the performance of diagnostic tests [43]. The area under the curve (AUC) was used as the outcome of RET.

Additionally, given that the ability of face recognition is considered a built-in visual expertise, the Cambridge Face Memory Test (CFMT)[40] was also employed to evaluate the level of face recognition ability for both groups (Fig. 1B). CFMT tasks are scored as number of trials correct out of 72 (i.e., accuracy for the total of all three stages). Note that chance = 24 trials (33.3% correct), given the three-alternative forced-choice response required on each trial [40].

### MRI data acquisition

Imaging data were collected using a 3Telsa MRI system (EXCITE, General Electric, Milwaukee, Wisc.) at the First Affiliated Hospital of Medical College, Xi’an Jiaotong University Xi’an, China. To eliminate the time-of-day effect, the scanning was performed from 9:00 a.m. to 11:30 a.m [44, 45]. A resting scan, a localizer scan, a structural and a DTI scan were conducted. The localizer data and DTI data were used for other studies and therefore not reported in this study. A standard birdcage head coil was used, along with restraining foam pads to constrain head motion and to reduce scanner noise.

For the fMRI scan, whole brain images were acquired with a gradient-echo single-shot echo planar imaging sequence. Parameters were: repetition time (TR) = 2 s; matrix = 64 × 64, field of view (FOV) = 240 mm; echo time (TE) = 30 ms. Thirty-two interleaved axial slices were oriented parallel to each participant’s anterior commissure-posterior commissure (AC-PC) line, with voxel size = 3.8 × 3.8 × 5.0 mm, gap = 0 mm. The fMRI scans lasted for 6 min and 20 s [46], resulting in 190 volumes. During the entire scan session, subjects were asked to keep their mind blank and keep their eyes open. After scanning, the subjects’ performance in the scanner were asked. Additionally, an MPRAGE T1-magnetization high resolution anatomical image (1 × 1 × 1 mm) was also acquired for each participant with the following parameters: TE = 2.26 ms, TR = 1900 ms, flip angle = 9°, FOV = 256 mm, slice thickness = 1 mm, matrix = 256 × 256. A total of 176 slices in the sagittal orientation were acquired. Potential clinical abnormalities of each participants were assessed by two expert radiologists based on the structural images. No participants were excluded at this level.

### Functional data preprocessing

Statistical Parametric Mapping (SPM12) (http://www.fil.ion.ucl.ac.uk/spm) and Data Processing Assistant for Resting-State fMRI (DPARSF) V2.4 advanced edition (http://www.restfmri.net/forum/DPARSF) [47] was used in the data preprocessing procedures under MATLAB2009a.

#### Resting data processing

The first 10 volumes of each subject were discarded to let the participants get adapted to the experimental environment and to only retain stabilized data. The images were preprocessed for slice timing, motion correction, co-registration to the subject’s anatomical images in native space. No subject was excluded for head motions, threshold was set at exceeding 1 mm of movement or 1°of rotation in any direction. Next, all the functional images were normalized to the MNI space and resampled to 3 mm isotropic voxels using the deformation field maps obtained from structural image segmentation, following the segmentation routine in SPM 12. After normalization, images were spatially smoothed with a 6 mm full width at half maximum Gaussian kernel. Finally, the linear trend was removed and temporal filtering (0.01–0.08 Hz [48, 49]) were performed on the time series of each voxel to reduce the effect of low-frequency drifts and high-frequency noise.

#### ALFF map

The ALFF analysis was carried out using the DPARSF v2.4, which has been described in previous studies [50]. Briefly, filtered time series (0.01–0.08 Hz) were transformed to the frequency domain using the fast Fourier transform. The square root was calculated at each frequency of the power spectrum and averaged across 0.01–0.08 Hz at each voxel and the ALFF metric was defined as the averaged square root. The ALFF of each voxel was further divided by the global mean ALFF value for each subject for standardization, as was the casein PET studies [51].

### Statistical analysis

#### Inter-group ALFF analysis

Statistical analysis was performed using SPM12. Voxel-wise comparison ALFF analysis was conducted across the whole brain. Two-sample t-test was performed to detect the ALFF difference between the two groups (RIG vs NECG). The significance level was set at cluster *p* < *0.05 after* multiple comparison correction (Alphasim corrected using Monte Carlo Simulations), with voxels uncorrected *p* < *0.00*1.

#### Correlation analysis

To investigate the relationship between the ALFF and behavior measurements (results of RET, CMFT, RT and cases reviewed in total) in the RIG, we computed the voxel-wise Pearson’s correlation analysis between ALFF and outcome of behavior tasks, i.e. CMFT, RET, RT of RET, as well as the duration of experience, i.e. cases reviewed in total. The significance level was set the same as Inter-group ALFF analysis.

#### Post hoc seed-based connectivity analysis

The mean BOLD time course of each ROI were extracted and a whole-brain seed-based functional connectivity analysis (FC) was conducted for each subject. The correlation coefficients were then converted to z scores using Fisher’s r-to-z transformation to obtain the entire brain z-score map of each subject. Two-sample t-test was performed to detect the connectivity differences between groups (RIG vs NECG) and the multiple comparison corrections was performed using the same Alphasim method as mentioned above.

## Results

### Results of behavior measurement

**Fig. 2:**
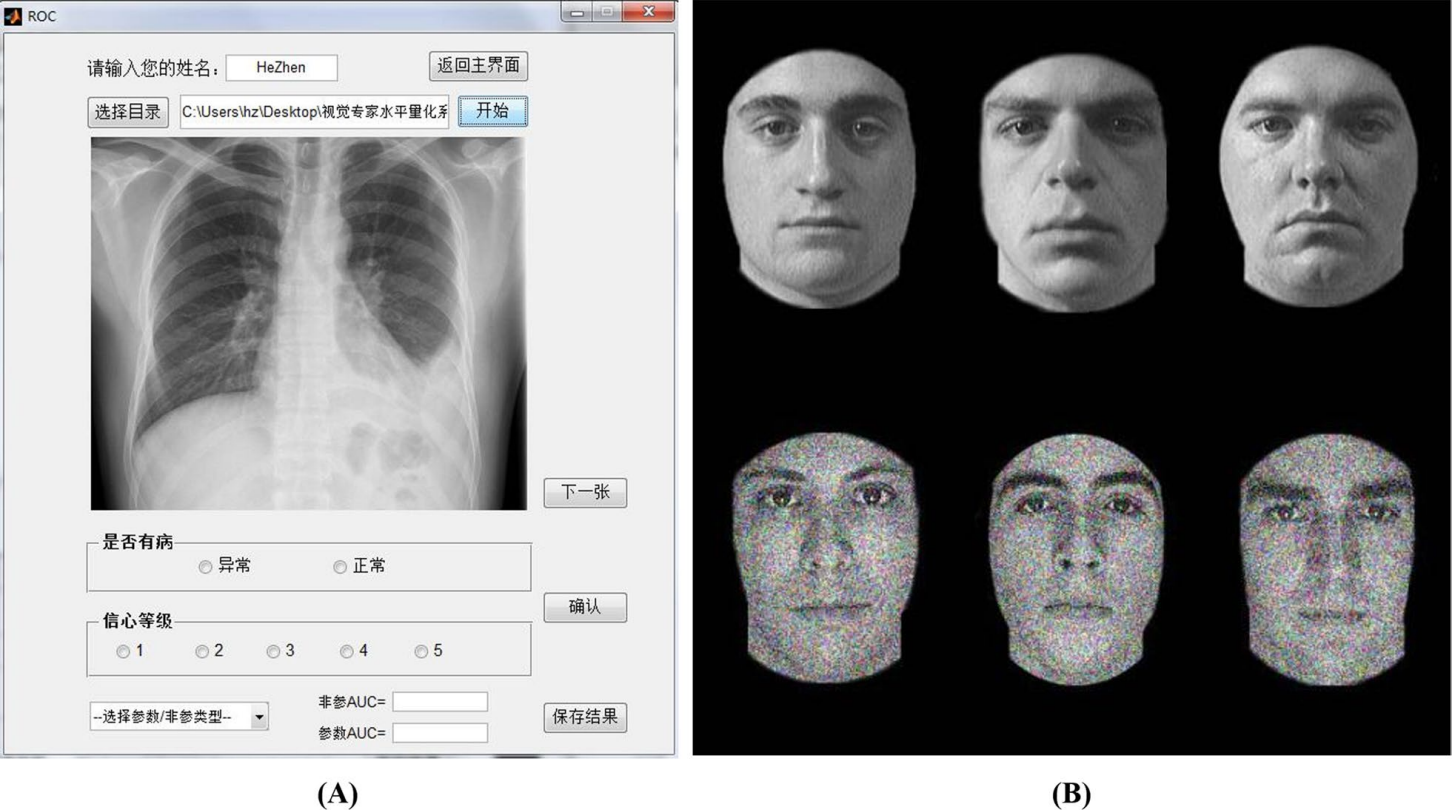
Samples of behavioral tests. **A** User interface of in-house software for the Radiological Expertise Task; **B** Stimulus used in Cambridge Face Memory Test

As shown in Table 1, this was no statistical difference in the level of visual expertise in face domain, as indicated by the results of Cambridge Face Memory Test (CFMT) between the RIG group and the non-expert control group (*p* = 0.28). As for the results of RET, the RIG group had a significantly higher AUC than the control group (*p* = 3.8 × 10^–22^, see Fig. 2A; Table 1 for details, Mann–Whitney test), indicating better visual recognition ability in the RIG. Moreover, for the RA group, the AUC of the ROC curve falls within the interval of 0.73–0.86. Following the guidelines of designing proper behavior tests for radiological performance [3], this interval shows that our experimental design is reliable. One month of radiological training in the X-ray department substantially increased their performance, which was illustrated by comments from their senior radiologists. This perceptual ability is obtained through training across review hundreds of cases [3, 6]. Moreover, although response time (RT) of RET was not used to determine the radiologists’ visual expertise in the clinical scenario, this parameter did reflect the behavioral expertise. The RIG was significantly faster in recognizing chest abnormalities than the control group (*p* = 1.3 × 10^–7^).

**Table 1:** The results of behavioral tests between the two groups

	Radiologists (n = 22)		Controls (n = 22)		*p*-Values
	Mean	SD	Mean	SD	
Length of training	26	2.4	–	–	–
Cases in Total	767.4	82.6	–	–	–
ROC(AUC)^a,b^	0.80	0.04	0.53	0.04	< .001
Response Time(s)	2.6	0.4	3.7	0.7	< .001
Face Expertise^a^	56.95	5.23	58.68	5.31	0.28

*AUC *area under curve, *ROC* Receiver operating characteristic, *SD* standard deviation, *s* seconds

^a^Denotes the item that shows significant difference between groups (*p* < *0.001*)

^b^Denotes that Mann–Whitney test was used

### Results of inter-group ALFF analysis

**Fig. 3:**
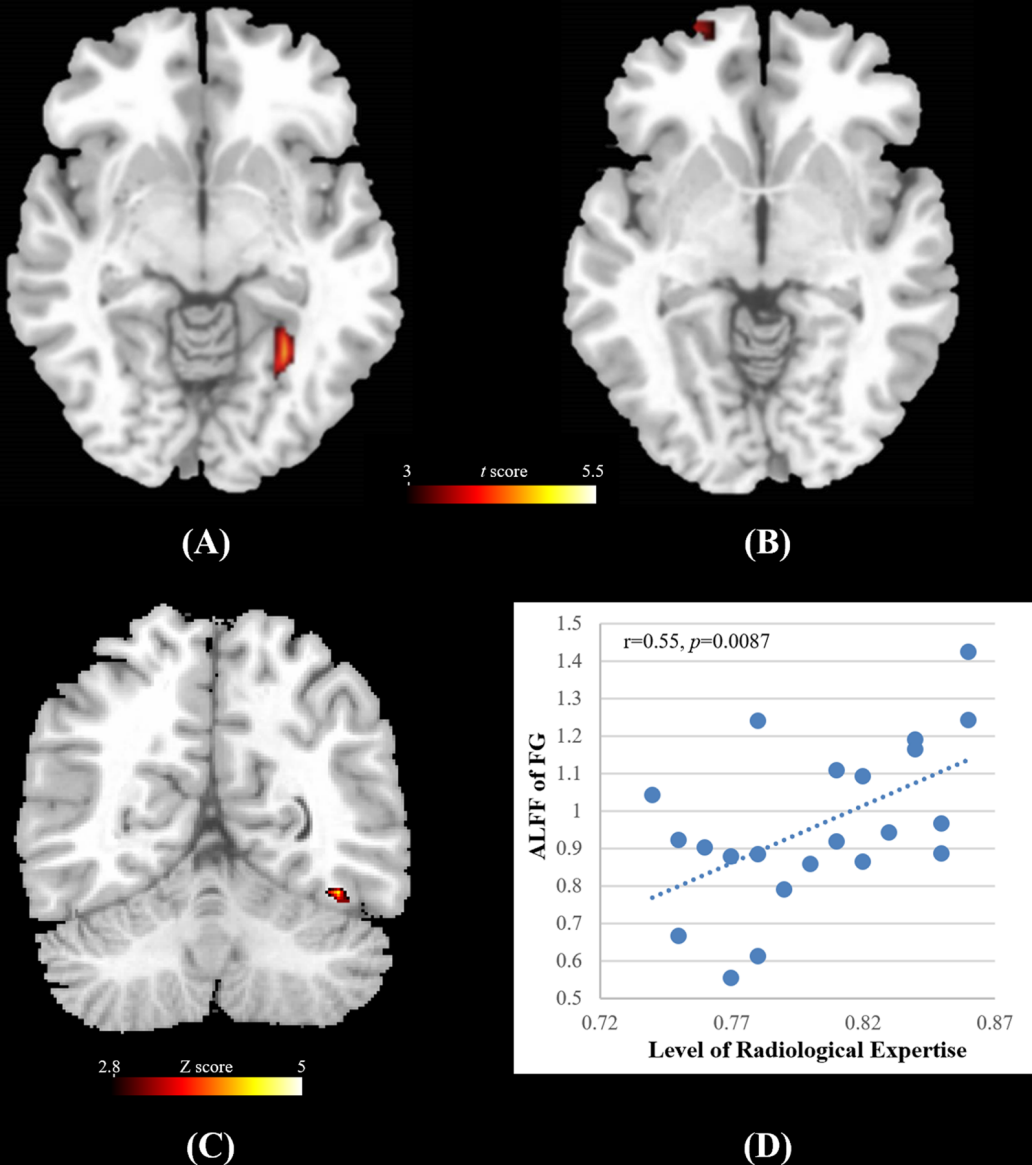
The ALFF differences between the radiology interns group (*n* = 22) and the normal control group (*n* = 22) (*p* < 0.05, alphasim corrected, *RIG v.s. NECG*) and voxel-wise correlation map between ALFF and the level of perceptual expertise in the domain of radiological images as assessed by the AUC of Radiological Expertise Task for the inter RIG group. **A** The intern radiologists group showed higher ALFF in the left OFC (displayed in sagittal view); **B** The intern radiologists group showed higher ALFF in the right fusiform gyrus (displayed in axial view); **C** Significant correlation between ALFF and visual recognition expertise was found the in the right fusiform gyrus (*p* < *0.05, multiple comparison corrected*); **D** The scatter plot map computed as ALFF of the peak voxel (40, 56, − 16) in the correlation analysis and RET scores. Please note that this map is only for illustration purpose, otherwise there would be the risk of a circular analysis. *OFC* the orbitofrontal cortex, *FG* the fusiform gyrus

Two-sample *t*-test results demonstrated a higher ALFF in the right fusiform gyrus (FG) and the left orbitofrontal cortex (OFC) in the RIG (*p* < *0.05, multiple comparison corrected*, Fig. 3A, B; Table 2). The loci of the FG is consistent with previous studies on fusiform face representation. No brain regions with a significant ALFF decrement were found.

**Table 2:** Peak activations of group ALFF differences between two groups (*p* < 0.05, multiple correction)

	Hemisphere	MNI Coordinates (cluster maxima)			Voxels	t (cluster maxima)	*p* (cluster maxima)
		x	y	z			
OFC	L	− 6	50	8	29	4.5	0.000
FG	R	58	− 12	− 25	16	5.2	0.000

*FG *the fusiform area, *OFC* the orbitofrontal cortex, *L *left, *R *right

### Results of the correlation analysis

A significant positive correlation between ALFF and the level of radiological expertise was found in the right FG in the RA group (*p* < 0.05, *r* = 0.55, Fig. 3C, D; Table 3). The MNI coordinate of peak voxel is (40, 56, -16). No significant correlations were found between outcomes of other behavior tests and ALFF in the RIG nor the NECG group.

**Table 3:** Significant voxel-wise correlation between ALFF and the level of perceptual expertise in the domain of radiological images as assessed by the AUC of Radiological Expertise Task in the RIG (*n* = 22) (*p* < 0.05, Alphasim corrected)

	Hemisphere	MNI Coordinates (cluster maxima)			Voxels	Z (cluster maxima)
		x	y	z		
FG	R	40	56	− 16	12	4.9

*FG *the fusiform area; R-right

### Post hoc seed-based connectivity analysis

Using FG and OFC as seed regions respectively, there was no significant group differences in connectivity between the FG and other brain regions nor OFC and other brain regions (*p* < *0.05, multiple comparison corrected*).

## Discussion

In past decades, behavior and cognitive studies endeavoring to understand radiologists’ expert visual recognition skills have achieved reliable scientific conclusions. Only recently, has attention been given to the neural substrate of such skill. Studies in this area of interest focused on the brain response during tasks; however, our current study focused on the restful brain by investigating a more ignored issue of how visual experience or expertise alters the level of intrinsic brain activity. The results of the behavior data analysis showed that the radiography interns group (RIG) significantly outperformed the non-expert control group (NECG) in radiological visual recognition tasks (Fig. 2; Table 1). The results of imaging data analysis showed a higher level of baseline brain activity, using ALFF as the metric, in the right fusiform gyrus (FG) and the left orbitofrontal cortex (OFC) in the RIG (*p* < 0.05, AlphaSim correction, Fig. 3A, B). Moreover, voxel-wise correlation analysis demonstrated that the level of visual recognition expertise correlated with the ALFF of FG in the RIG (Fig. 3C,D). The current study is the first to investigate the focal feature of radiologists’ resting brain by elevating the level of intrinsic brain activity changes. Given that resting-state brain activity is the sum of previous experience [27], we proposed that these alterations may represent the visual experience in radiological interpretation and participate in skill maintenance.

Our results elucidated higher ALFF in the right FG of the RIG. The FG is constantly reported in task fMRI studies using visual expertise models of other domains, such as cars [10, 52], birds [53], chess [54] and faces [55]. It plays a vital role in visual categorization learning [56, 57]. Specifically, FG process higher-level visual information [12] and is involved in fine-grained visual recognition independent of the categories of visual stimuli, either for real-life or lab-based objects [14, 41, 58]. Its activity was positively correlated with participants' perceptual performance [59–61] and could be modulated by visual learning [57]. Previous conclusions suggest that both enriched sensory input and training lead to improved perceptual performance, which is in parallel with both structural and functional plastic changes [62]. Additionally, the level of visual expertise in the domain of radiological images correlated with the ALFF in this region (Fig. 3C). We proposed that alterations in the fusiform gyrus likely play a pivotal role in supporting perceptual proficiency, which is illustrated by intern radiologists’ better behavior performance in the RET (Table 1; Fig. 2). A recent study reported that the volume of regional cerebral blood flow was correlated with ALFF in the brain region from the resting state data [38]. Previous PET studies reported an increase in cerebral blood flow after sensorimotor learning in the restful human brain [63], indicating excitability in neuronal activities. Taken together, we propose that learning and clustered changes in a specific region are likely to be associated with higher ALFF values. In other words, higher ALFF in the right FG may indicate the specialization of sensory cortices in support of perceptual awareness in a given modality [64], which may further facilitate increased processing of visual stimuli in radiologists [28, 65]. We suggest that the ALFF difference in the FG between groups is likely to be driven by intensive learning experience with radiological image interpretation, given that expertise in other domains was excluded from subject inclusion and the difference in face expertise was also controlled (Fig. 2C). But, further studies with longitudinal experimental design are encouraged to answer the question how short-term training in radiological image interpretation modulates visual experience. Nevertheless, it should be noted that The FG is the most reported brain region engaged in many domains of visual expertise [53], such as faces, cars [66], birds^53^, chess [67], musical notes^68^ and etc. Given the cross-sectional design employed by the current study, it is possible that the result is attributed to other kinds of visual expertise, although the known domain of visual expertise is controlled. Further study using longitudinal experimental design can add extra line evidence to this issue.

In addition, higher ALFF was found in the left OFC of the RIG than that of the NECG. Without further support from data analysis ("MRI data acquisition" section Post hoc seed-based connectivity analysis), we could only speculate the potential role of the left OFC in medical image interpretation. For the radiologists, their visual search is guided by the high-speed mechanism, i.e., fast holistic searching mode, rather than search-to-find mode [69], which established the expert impression of the gestalt of an image, resulting in immediate understanding of the gist of a medical image despite its dramatic complexity and ambiguity [70]. This brief process significantly decreases the temporal and computational load required for object recognition [71]. This was supported by the results of behavioral analysis that the RIG was significantly faster in recognizing medical images (Fig. 2B; Table 1). The OFG uses coarsely-analyzed information to generate a gist of perceptual decisions about possible locations for further fine-grained identification[71, 72] and guide subsequent visual search procedures [73], which facilitates visual recognition. Moreover, a previous resting state MRI study demonstrated that training-induced skill acquisition would optimize interregional communication efficiency in the participants [74]. We suggest that clustered changes in the left OFC, as indicated by the higher ALFF, is coherent with this idea and may reflect a tendency that facilitates behavioral expertise. Taken together, we speculate that the increased baseline brain activity in the left OFC is likely to contribute to the holistic searching process. Again, without further support, it should be emphasized that the role of the OFC should be specified by additional evidence from future studies [8, 12, 69–75].

## Limitation

Several limitations should be taken into consideration for the current study. First, the sample sizes is comparatively small in the current study given the rigorous subject screening procedures to control the homogeneity and confounding factors of both groups. The findings are expected to be replicated by our subsequent studies using larger sample sizes. Second, it would be ideal if the NECG consisted of interns in a different medical program other than not radiology. Third, akin to all cross-sectional experimental designs, the observed higher baseline brain activity is likely to be attributed to one-month of training in the X-ray department and other confounding factors, such as training in critical periods during development or genetic predisposition, which are unlikely to be eliminated in a cross-sectional experimental design. Taken together, a longitudinal design in which ALFF before and after radiological training was assessed would alleviate all these concern. Interpretations of current findings should take these issues into consideration.

## Conclusion

Our current study provides the first evidence of how visual experience/expertise modulates baseline brain activity in the resting stateIt may shed light on the development of visual recognition skills in medical image interpretation by illustrating the engagement of both top-down and bottom-up processes. We hope that by revealing the neural mechanism of radiological visual expertise, more efficient education strategies can be developed [76].

## Data Availability

The datasets used and/or analyzed during the current study are available from the corresponding authors on reasonable request.

## Abbreviations

AC-PC: Anterior commissure-posterior commissure
ALFF: Amplitude of low-frequency fluctuation
AUC: Area under curve
BOLD: Blood oxygen level-dependent
CFMT: Cambridge Face Memory Test
DPARSF: Data Processing Assistant for Resting-State fMRI
FFT: Fast Fourier transform
FG: Fusiform Gyrus
fMRI: Functional Magnetic Resonance Imaging
FOV: Field of view
NECG: Non-expert control group
OFC: Orbitofrontal cortex
PACS: Picture Archiving and Communication System
RET: Radiological Expertise Task
RIG: Radiology interns group
ROC: Receiver operating characteristic
SPM: Statistical Parameter Mapping
TE: Echo time
TR: Repetition time
